# Hsrω and Other lncRNAs in Neuronal Functions and Disorders in *Drosophila*

**DOI:** 10.3390/life13010017

**Published:** 2022-12-21

**Authors:** Anand Kumar Singh

**Affiliations:** Interdisciplinary School of Life Sciences, Institute of Science, Banaras Hindu University, Varanasi 221005, India; anandksingh@bhu.ac.in

**Keywords:** LncRNA, hnRNP, RNA processing, neurodegeneration, polyQ, ALS, Drosophila

## Abstract

Long noncoding RNAs (lncRNAs) have a crucial role in epigenetic, transcriptional and posttranscriptional regulation of gene expression. Many of these regulatory lncRNAs, such as MALAT1, NEAT1, HOTAIR, etc., are associated with different neurodegenerative diseases in humans. The lncRNAs produced by the *hsrω* gene are known to modulate neurotoxicity in polyQ and amyotrophic lateral sclerosis disease models of *Drosophila*. Elevated expression of hsrω lncRNAs exaggerates, while their genetic depletion through *hsrω-RNAi* or in an *hsrω*-null mutant background suppresses, the disease pathogenicity. This review discusses the possible mechanistic details and implications of the functions of hsrω lncRNAs in the modulation of neurodegenerative diseases.

## 1. Introduction

Long noncoding RNAs (lncRNAs) have well-programmed expression profiles in neurons, and many of them are now known to be associated with human neurodegenerative diseases [1,2,3,4]. LncRNAs impact neurodegeneration by regulating multiple cellular activities such as chromatin organization, epigenetic regulation, gene expression or signaling pathways. Some of the neurodegeneration associated with lncRNAs sequester RNA processing proteins to form liquid–liquid phase separation (LLPS)-based, membraneless structures which directly or indirectly affect widespread gene expression. For example, the ubiquitously expressing nuclear-enriched abundant transcript 1 (NEAT1) lncRNA in human recruits several RNA-binding proteins (RBPs) from the Drosophila behavior/human splicing (DBHS) protein family, such as PSF/SFPQ, NONO/P54NRB and PSPC1, to organize the paraspeckles, which act as molecular hubs for different cellular processes and whose misregulation can lead to neurodegeneration. [5,6,7]. Likewise, the abundant lncRNA metastasis-associated lung adenocarcinoma transcript 1 (MALAT1), also known as NEAT2, binds with a specific set of mRNA-processing proteins to form nuclear splicing speckles and interacts with *miR-125b* to inhibit neuronal apoptosis and suppress inflammatory cytokines in Alzheimer’s disease [8,9].

The present review briefly discusses the roles of lncRNAs associated with neurological disorders in *Drosophila melanogaster*, with a focus on the hsrω lncRNAs. Similar to NEAT1 and MALAT1 lncRNAs, the hsrω lncRNAs in *Drosophila* and their functional analog, Sat III lncRNAs in humans, sequester a large set of RBPs to control transcription and posttranscriptional processing of mRNAs. The hsrω lncRNAs modulate the neurotoxicity of diseases associated with abnormal RNA processing such as amyotrophic lateral sclerosis (ALS) and polyQ. Likewise, Sat III lncRNA is associated with the frontal cortex in frontotemporal lobar degeneration (FTLD) disease in humans. Based on experimental evidence, this review considers the possible mechanistic understanding of involvement of hsrω lncRNAs in different fly models of human neurodegenerative diseases.

## 2. LncRNAs Associated with Neuronal Functions and Diseases in *Drosophila*

*Drosophila* has been widely used as a model system to examine the involvement of different genes including lncRNAs in brain development and neurodegenerative diseases. The neuron-specific lncRNA *CASK regulatory gene* (*CRG*) was found to control locomotory behavior in *Drosophila* by upregulating the expression of its neighboring gene, *CASK* [10], which is a protein-coding gene involved in walking behavior in *Drosophila* [11,12]. It is suggested that the *CRG* lncRNA upregulates *CASK* expression by facilitating the association of RNA Pol II transcription machinery at the promoter region of the *CASK* gene [10]. Further studies are required to understand the molecular mechanism and significance of *CRG*-dependent regulation of *CASK* expression in *Drosophila*. The lncRNA gene *yellow-achaete intergenic RNA* (*Yar*) is located within a neural gene cluster of *Drosophila*, and loss of *Yar* lncRNA in Yar-null mutants causes shortened nighttime sleep bouts within a normal circadian sleep–wake cycle and down-regulates levels of sleep rebound following deprivation [13]. These sleeping abnormalities are rescued by the transgenic expression of *Yar*, which demonstrates that *Yar* is indeed required for sleep regulation in *Drosophila* [13,14]. Although the specific mechanism of regulation of the sleeping behavior in *Drosophila* by *Yar* is not clear, it is speculated to affect the stabilization or translation of a wide range of cytoplasmic mRNAs in the cell. The lncRNA produced by the *iab-8* gene represses *abd-A* expression in neural cells by either producing a microRNA (*miR-iab-8*) or by transcriptional interference of RNA pol II at the 3′ end of the *iab-8* gene, which overlaps with the *abd-A* promoter [15]. Loss of *iab-8* lncRNA causes sterility in both male and female flies, inducing behavioral defects. Knocking down *iab-8* lncRNA expression affects the bending of the male abdomen and thereby prevents copulation with female flies, while female flies with knocked down *iab-8* lncRNA cannot pass eggs through the oviduct [15].

A genetic screen for identifying the genes that interact with *dFIG4*, one of the causative genes for Charcot–Marie–Tooth disease (CMT), identified *CR18854* lncRNA as a suppressor of CMT phenotypes in *Drosophila* [16,17]. The *CR18854* gene, located at the 30D1 site, encodes a 2566 base-long hairpin RNA that generates endogenous short-interfering RNA [18]. The *CR18854* lncRNA binds with Hrp59 and Staufen RBPs, and thus has widespread effects on gene expression at the post-transcriptional level. Loss of *CR18854* lncRNA suppresses the enlarged lysosome phenotype induced by the fat body-specific knockdown of *dFIG4* [18]. The *CR18854* gene also shows a genetic interaction with Cabeza (dFUS), a *Drosophila* homolog of human FUS and one of the causing genes of amyotrophic lateral sclerosis (ALS) [18]. Another lncRNA, *CR43467,* was also identified as a suppressor of the rough-eye phenotype of dFIG4 and as a rescuer of the enlarged lysosome phenotype induced by fat body-specific knockdown of dFIG4 [19]. The lncRNAs of the *hsrω* gene also interact with the dFIG4 in neurons, which suggests that *CR18854* lncRNAs and hsrω lncRNAs may interact with common factors such as Cabeza in CMT and ALS pathogenesis [16,17].

## 3. Role of Hsrω lncRNAs in Neuron Development and Neurodegenerative Diseases

**Figure 1:**
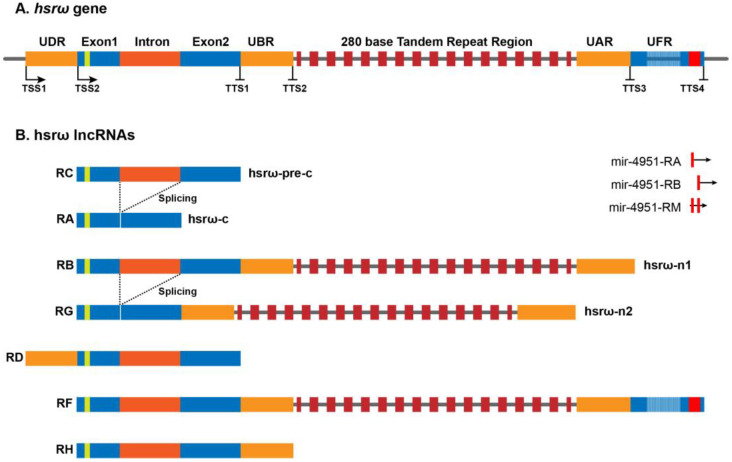
Architecture of the *hsrω* gene and its seven long noncoding transcripts in *Drosophila melanogaster* (http://flybase.org/reports/FBgn0001234, accessed on 12 December 2022). (**A**) shows different parts of the *hsrω* gene including Exon1, Intron, Exon2, tandem repeats of 280 bases and the different unique regions [21]. The yellow box in Exon 1 represents the short open reading frame (sORF) while the red box in UFR indicates a microRNA gene (*mir-4951*). (**B**) shows the seven known hsrω lncRNAs and three microRNAs, *mir-4951-RA*, *mir-4951-RB* and *mir-4951-RM*, transcribed from the *mir-4951* gene (redrawn after http://flybase.org/reports/FBgn0001234, accessed on 12 December 2022).

The heat-shock RNA omega (hsrω) is a developmentally active, heat-shock inducible, noncoding gene conserved in all the known species of *Drosophila* [20,21,22]. Originally, the *hsrω* gene was believed to be composed of two exons, E1 (~475 bp) and E2 (~750 bp), separated by an intron (~700 bp), followed by a long stretch of 280 bp of tandem repeats extending for ~5 kb to ~15 kb of length (Figure 1) [23,24], and it was believed to produce two primary transcripts, hsrω-n1 and hsrω-pre-c, after splicing form hsrω-n2 (nuclear) and hsrω-c (cytoplasmic) transcripts, respectively [23,25,26,27]. The hsrω-c transcript localizes to cytoplasm while the hsrω-n1, hsrω-n2 and hsrω-pre-c transcripts remain confined to the nucleus. The hsrω-c, hsrω-n1, hsrω-n2 and hsrω-pre-c have been renamed at the FlyBase (www.flybase.org) as hsrω-RA, hsrω-RB, hsrω-RG and hsrω-RC, respectively (Figure 1). Based on the high throughput sequencing data, FlyBase has added three additional RNAs (hsrω-RD, hsrω-RF and hsrω-RH) and three miRNAs to the list of noncoding RNAs produced by the *hsrω* gene (Figure 1). Further investigations on the expression profiles and functions of these newly identified hsrω transcripts are required for a better understanding of the functions and significance of the *hsrω* gene and its conservation in *Drosophila* species.

Most studies on the *hsrω* gene have so far focused primarily on functions of the 280 bp repeats containing nucleus-restricted hsrω-n lncRNAs (hsrω-RB and hsrω-RG); since there has been no specific information on the longest hsrω-RF transcript, it is not known if its functions overlap with those of the hsrω-RB and hsrω-RG transcripts. Since the 280 bp repeats have been found to be localized by in-situ hybridization, either in the omega speckles or at the site of transcription [27,28,29,30,31], it may be presumed that the hsrω-RF transcripts are also present in the omega speckles. Accordingly, the hsrω-RB, hsrω-RG and hsrω-RF transcripts are referred together as hsrω-n lncRNAs. The hsrω-n lncRNAs sequester multiple hnRNPs, chromatin remodeling factors, components of the nuclear membrane, and some disease-associated RBPs (Table 1) to form the nucleoplasmic omega speckles [27,28,29,30,31,32]. The ~1.9 kb long nuclear hsrω-pre-c lncRNA is spliced to remove the ~700 b long omega intron to produce the ~1.2 kb cytoplasmic hsrω-c (hsrω-RA) lncRNA. The hsrω lncRNAs carry a potentially translatable 23–27 amino-acid-long open reading frame (ORFω) in Exon 1 [21,23,33]. Although the sequence of ORFω is divergent, the organization of exons, ORFω and the omega intron of the *hsrω* gene is highly conserved in different species of *Drosophila*, which suggests that either translational activity of hsrω-c or the small omega peptide product may have an important role in cellular functions [21]. A systemic study on cellular functions of hsrω-c lncRNA and its omega peptide will help to explore the absolute functional potential of the *hsrω* gene in *Drosophila*.

Since *hsrω* is a heat-shock-inducible gene, initial studies were based on the characterization of *hsrω* gene expression regulation under various cell stress conditions. RNA:RNA in situ hybridization studies suggest that hsrω lncRNAs are ubiquitously expressed with a tissue-specific variation in their levels [30,46,47]. The hsrω lncRNAs play an important role in maintaining the cell physiology as mutants with disrupted hsrω lncRNA functions, as in *hsrω-null*, *hsrω-RNAi*, *ISWI-null* or *Hrb87F-null* show delayed development, developmental lethality, short life span, reduced fecundity and reduced stress tolerance [26,31,40,48,49]. Following the discovery of a central role of these transcripts in sequestering RBPs in omega speckles, functions of hsrω lncRNAs were investigated in different biological processes that are influenced by abnormal functions of RBPs. Since abnormal functions of RBPs severely affect neuronal development and function, the roles of hsrω lncRNAs have been studied in different neurodegenerative diseases such as polyQ and ALS.

## 4. Role of Hsrω lncRNA in polyQ Expansion Disorders

The first indication of a potential role of hsrω lncRNAs in neurodegeneration was obtained in a genetic screen of the factors that regulate spinocerebellar ataxia type 1 (SCA-1) neurotoxicity in *Drosophila* [50]. SCA-1 is a neurodegenerative disease caused by the expansion of the polyglutamine (polyQ) tract in the ataxin-1 protein. In a genetic screen, two mutant alleles of the *hsrω* gene, *hsrω^05241^* and *P292,* were identified as enhancers of SCA-1-induced neurotoxicity [50]. Afterwards, UAS/GAL4-based overexpression alleles of the *hsrω* gene, viz. *EP3037* and *EP93D,* were also reported to enhance the neurodegeneration caused by the expression of expanded polyQ (127Q) or of expanded huntingtin protein (Htt-ex1p-93Q) in the developing eyes of *Drosophila* [51]. Interestingly, a null allele of Hrb87F, an hnRNP associated with hsrω-n lncRNA in omega speckles (Table 1), also dominantly enhanced the neurodegeneration. Despite the strong genetic interaction between the *hsrω* gene and the polyQ-expanded transgene, neither hsrω-n lncRNA nor the associated hnRNPs were found to display any distinct colocalization with the polyQ inclusion bodies in *Drosophila* eye disc cells [51]. Further studies revealed that the RNAi-based selective depletion of hsrω-*n* lncRNAs using eye-specific *GMR-GAL4* drivers dramatically suppressed the polyQ pathogenesis and restored pigmentation and ommatidial arrays in adult eyes [52]. Loss of hsrω-n lncRNA suppressed eye-specific degeneration in a variety of expanded polyQ backgrounds such as the 127Q, ataxin-1 Q82 (SCA1), MJDTR-Q78 (SCA3) or Httex1p Q93 (Huntington’s disease) fly models [52]. The RNAi-mediated depletion of hsrω lncRNA reduced the polyQ aggregates without reducing the mutant mRNA levels, which suggests that hsrω lncRNAs affect either the polyQ mRNA translation or polyQ protein stability. Interestingly, down-regulation of hsrω-n transcripts had only a marginal effect on neuropathy caused by the over-expression of wild-type or mutant tau protein in flies [52], suggesting a selective role of these lncRNAs in modulating neurodegeneration [53]. Modulation of activities of the CREB-binding protein (CBP) and the *Drosophila* inhibitor of apoptosis protein 1 (DIAP1) by the hsrω lncRNAs have been suggested to be some of the causal factors that ameliorate polyQ toxicity [54].

## 5. Role of Hsrω lncRNAs in ALS Disease

Amyotrophic lateral sclerosis (ALS) is one of the most common adult-onset motor neuron diseases in which both upper and lower motor neurons have a progressive loss that results in muscle weakness, paralysis and premature death. Although 90–95% of ALS cases are sporadic and only 5–10% are familial, most of them have alterations in RNA metabolism. ALS is a clinical outcome of the malfunctioning of different RNA-binding proteins (RBPs) including FUS, C9ORF72, TDP-43, hnRNPA1, ATXN2, ANG and TAF15 [55,56,57]. The dysfunctional forms of these proteins get mislocalized in the cytoplasm and form pathogenic aggregates. Loss of hsrω lncRNAs in motor neurons also causes ALS-like phenotypes in *Drosophila* [36]. The pan-neuronal *ELAV-GAL4*-driven RNAi-based depletion of hsrω-n transcripts induces anatomical defects in presynaptic terminals of motor neurons that impair locomotion in larval as well as adult flies and shorten their life span [36]. Similarly, the motor neuron-specific *D42-GAL4*-driven loss of hsrω lncRNA reduces the terminal synapse branch length, branch number and boutons number at neuromuscular junctions (NMJ), which suggests that hsrω lncRNAs have an important role in the development of NMJ in *Drosophila*.

The Cabeza or dFUS protein of *Drosophila* is a homolog of the human FUS protein. FUS is an hnRNP with diverse roles in transcriptional and post-transcriptional regulation of gene expression [58]. Similar to many RBPs, the Cabeza also binds with hsrω lncRNAs in omega speckles, and depletion of hsrω lncRNA enhances its cytoplasmic localization leading to loss of Cabeza functions in the nucleus [36]. This suggests that hsrω-n lncRNAs in omega speckles act as nuclear anchors for Cabeza and thus facilitate Cabeza’s nuclear functions. The role of hsrω lncRNAs in the ALS model of *Drosophila* was further investigated by a transgenic expression of FUS. FUS is largely a soluble protein but forms cytoplasmic inclusion bodies in a range of FUS proteinopathies such as ALS-FUS in motor neuron disease or FTLD-FUS in frontotemporal lobar degeneration, etc. The FUS-induced neurotoxicity in ALS is rescued by RNAi-mediated depletion of hsrω lncRNAs in *Drosophila* [59]. The GMR-GAL4-driven FUS expression in *Drosophila* optical neurons induces loss of pigmentation with aberrant eye morphology. These phenotypes were reversed by loss of hsrω lncRNAs, which resulted in the elimination of soluble FUS aggregates through the formation of cytoplasmic, nontoxic, insoluble inclusions of FUS-LAMP1 (lysosome-associated membrane protein 1) [59]. LAMP1 is the most abundant protein on the lysosome membrane and is used as a marker of autophagy. The insoluble inclusions of FUS-LAMP1 in hsrω-lncRNA-depleted cells are suggested to be degraded via autophagy [59]. This suggests a novel function of hsrω-n lncRNA in sequestering the toxic FUS protein from a soluble state to a harmless insoluble aggregate. To understand how hsrω regulates the localization and stability of FUS, arginine methylation of FUS, which is known to control its cellular localization and/or solubility, was investigated [60,61]. The hsrω lncRNAs were found to regulate the abundance of both type I and type II *Drosophila* arginine methyltransferases (DARTs) that control the methylation of FUS [62]. The arginine demethylation of FUS by DART1 and DART5 is the fundamental modification underlying the *hsrω*-knockdown-dependent suppression of FUS toxicity. However, how hsrω lncRNA regulates the expression of DARTs awaits further investigation.

The neurotoxicity caused by ALS-inducing factor TAR DNA-binding protein 43 (TDP-43) is also modulated by hsrω lncRNA levels. TDP-43 is a major protein associated with inclusion bodies in ALS and frontotemporal lobar degeneration (FTLD-TDP) [63]. The transgenically expressed TDP-43 binds with hsrω lncRNA in omega speckles and accumulates at the *hsrω* gene site, the 93D locus, during heat shock [37,38]. The level of hsrω lncRNA is upregulated ~two-fold in TDP-43 over-expressing neurons, and loss of hsrω lncRNAs in *hsrω-RNAi* partially mitigates TDP-43 over-expression-associated neurodegeneration in eyes [37]. Studies based on the *Drosophila* polytene chromosomes and high-throughput ChIP-seq suggest that TDP-43 targets RNA Pol II super elongation complex (SEC) components ELL and Lilli at the *hsrω* gene site to elevate its expression [37,38]. This suggests that hsrω lncRNAs are functional targets of TDP-43, and therefore, altered expression of hsrω modulates TDP-43-mediated neurodegeneration. It would be interesting to examine the roles of SEC factors in the induction of hsrω lncRNA expression in polyQ or FUS-ALS disorders.

## 6. Hsrω lncRNAs as a Hub for RBPs to Modulate Neurodegeneration

Based on the experimental evidence, hsrω lncRNAs emerged as modulators of neurodegenerative diseases that are caused by abnormal RNA processing such as polyQ, FUS-ALS or TDP43-ALS. The RNAi-mediated depletion of hsrω-n lncRNAs upregulated the association of hnRNPs with CBP, enhanced the level of DIAP1 through its association with Hrb57A hnRNP and improved the proteasomal activity in polyQ diseases [28,52,53,54,64]. Likewise, the loss of hsrω lncRNAs also enhanced the FUS–LAMP1 interaction and DARTs expression in FUS-ALS disease [36,65]. The suggestion that hsrω-n lncRNAs act as a hub for regulating the cellular dynamics of different proteins associated with RNA metabolism [28,29,31] may explain how hsrω-n lncRNAs affect localization, interactions and functions of client proteins. The mechanistic details of these events are still unknown.

**Figure 2:**
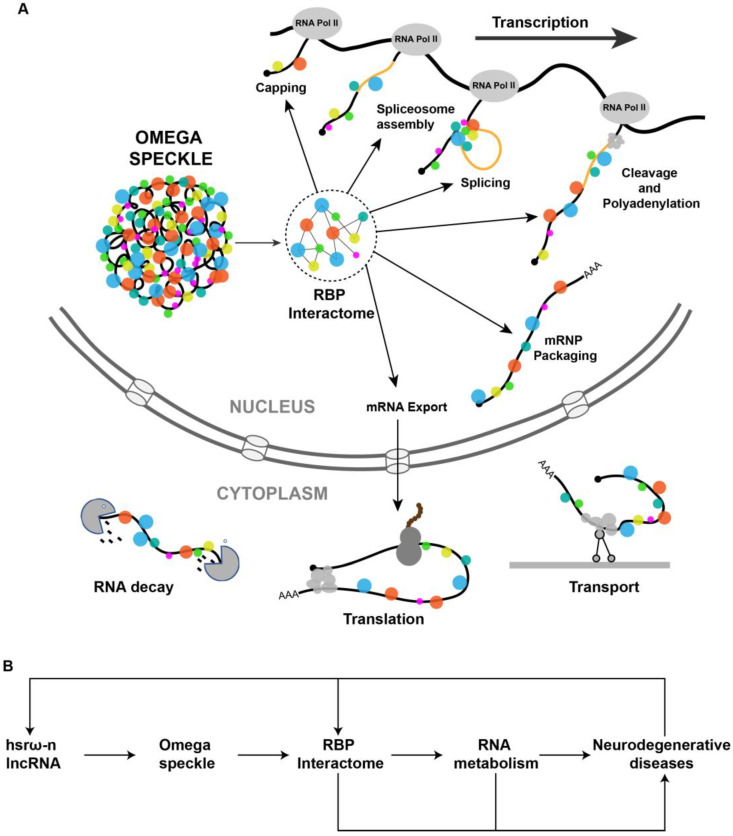
(**A**) Pictorial view of omega speckles’ functions in modulating co-transcriptional mRNA-processing events such as capping, splicing, cleavage and polyadenylation, mRNP Packaging to mRNA export, localization, translation and mRNA decay. (**B**) A simplified schematic diagram showing the role of hsrω lncRNAs and omega speckles in the regulation of neurodegenerative disease by modulating the nuclear RBP interactome and RNA metabolism.

The hsrω-n lncRNAs associated RBPs in omega speckles are involved in different steps of mRNA processing including splicing, export, localization, stability and translation (Figure 2A) [27,49,66,67,68]. The omega speckles are not formed following the loss of hsrω-n lncRNAs through *hsrω-RNAi* or in *hsrω-null* mutants or due to the absence of Hrb87F (Hrp36) hnRNP in *Hrb87F-null* flies [27,29]. Under these conditions, the omega speckle-associated RBPs get released in the nucleoplasm leading to an increase in the pool of free RBPs in functional compartments [31,53]. This increase in the titer of free RBPs is suggested to change the RBPs’ interactome in the nucleoplasm [28,29,31], which would ultimately globally modulate the RNA metabolism in the cell (Figure 2B). The primary cause of ALS is a reduction in the amount of functionally active forms of FUS or TDP-43 in the nucleus. Loss of hsrω-n lncRNA releases these RBPs from omega speckles so that improvement in their availability for nuclear RNA processing rescues the neurotoxicity. Regulation of the demethylation of FUS by hsrω lncRNAs [62] is another path through which these lncRNAs affect neurotoxicity (Figure 2B). These studies suggest that hsrω-n lncRNAs act at multiple levels to modulate the functions of RBPs in neurodegenerative diseases.

## 7. Future Perspectives

The current understanding is that hsrω-n lncRNAs modulate neurodegenerative disorders by regulating the dynamic availability of diverse RBPs associated with omega-speckles (Table 1). RBPs are essential for maintaining the transcriptome through their wide-ranging effects on pre-mRNA processing, mRNA transport, localization, translation and the decay of RNAs in the cell. Abnormal functions of RBPs and consequent widespread defects in RNA processing are common pathogenic underpinnings for several neurological disorders such as spinal muscular atrophy (SMA), Alzheimer’s disease (AD), amyotrophic lateral sclerosis (ALS), frontotemporal dementia (FTD), multiple sclerosis (MS), congenital myasthenic syndrome (CMS) and fragile X-associated tremor/ataxia syndrome (FXTAS) [69,70,71,72,73].

A comprehensive list of proteins associated with omega speckle remains unknown. A systematic molecular approach to characterize the omega-speckle components may unravel some novel hsrω lncRNA-associated factors or pathways with roles in neuronal development and differentiation. Likewise, the roles of hsrω lncRNAs, other than the nuclear ones, in neurodegeneration are still unknown. A systematic study to examine the expression and functions of all seven known lncRNAs of the *hsrω* gene in neurodegenerative diseases will provide a comprehensive information about the diverse hsrω lncRNAs in RBP-associated neurodegenerative diseases.

Studies on hsrω lncRNA would also be useful to explore the therapeutic potential of Sat III lncRNA, a human functional analogue of the *Drosophila* hsrω lncRNA [35], which also shows elevated expression in TDP-43-mediated FTLD disease [37]. Sat III lncRNA also accumulates RBPs such as HSF1, HAP, CBP, Sam68, C2PA, 9G8, ASF/SF2 and SRp30 at the site of their transcription (9q12 at chromosome 9) during heat-shock treatment to form the nuclear stress bodies (nSBs) in human cells [74]. This indicates a similarity in functions of omega speckles and nSBs in sequestering different RBPs during stress [35,75]. The underlying mechanistic details about factors and pathways that regulate the expression and functions of hsrω lncRNAs in ALS will help in understanding the role of Sat III lncRNA in human ALS pathogenesis. Thus hsrω lncRNAs can be used as potential models to understand the mechanism through which lncRNAs are involved in either the establishment and progression or the rescue of various diseases and developmental defects.

## Figures and Tables

**Table 1:** List of proteins known to be associated with hsrω-n lncRNAs in Omega speckles.

Name/Synonyms	Mammalian Homolog	Spatial Location and Other Features	References
Hrb87F/Hrp36	hnRNPA1	hnRNP, present in omega speckles and accumulates at *hsrω* gene site (93D) during heat shock	[27,30,31]
Hrb98DE/Hrp38	hnRNP A	hnRNP, present in omega speckles and accumulates at *hsrω* gene site (93D) during heat shock	[27,30,31]
Squid/Hrp40	hnRNP D	hnRNP, present in omega speckles and accumulates at *hsrω* gene site (93D) during heat shock	[27,30,31]
Hrb57A/Bancal	hnRNP K	hnRNP, present in omega speckles and accumulates at *hsrω* gene site (93D) during heat shock	[30,31,34]
Rumpelstiltskin/Hrp59	hnRNP M	hnRNP, present in omega speckles and accumulates at *hsrω* gene site (93D) during heat shock	[30,35]
Cabeza	FUS	RNA-binding protein implicated in ALS, present in omega speckles	[36]
TDPH	TDP-43	RNA-binding protein implicated in ALS, present in omega speckles and accumulates at *hsrω* gene site (93D) during heat shock	[37,38]
NonA	P54NRB, PSPC1, PSF	DBHS (*Drosophila* behavior and human splicing) family proteins, present in omega speckles	[27,39,40]
PEP	ZNF236	Preferentially present on ecdysone-induced sites, also present in omega speckles	[30,35]
Sxl	HuR	Involved in sex determination and dosage compensation, present in omega speckles	[30,41]
ISWI	SMARCA1	Catalytic sub-unit of chromatin remodeling complex transitionally localized in omega speckles, essential for omega speckle maturation and localization	[40,42]
Hsp83	Hsp90	Heat-shock protein, present in omega speckles and accumulates at *hsrω* gene site (93D) during heat shock	[43] Fatima and Lakhotia, Unpublished
SAF-B	SAF-B	Nuclear matrix protein, present in omega speckles and accumulates at *hsrω* gene site (93D) during heat shock	[31,44]
Megator	TPR	Nuclear matrix protein, present in omega speckles and accumulates at *hsrω* gene site (93D) during heat shock	[31,45]

## References

[1] Riva P., Ratti A., Venturin M. (2016). The Long Non-Coding RNAs in Neurodegenerative Diseases: Novel Mechanisms of Pathogenesis. Curr. Alzheimer Res..

[2] Roberts T.C., Morris K.V., Wood M.J. (2014). The role of long non-coding RNAs in neurodevelopment, brain function and neurological disease. Philos. Trans. R. Soc. Lond. B Biol. Sci..

[3] Wan P., Su W., Zhuo Y. (2017). The Role of Long Noncoding RNAs in Neurodegenerative Diseases. Mol. Neurobiol..

[4] Zhang M., He P., Bian Z. (2021). Long Noncoding RNAs in Neurodegenerative Diseases: Pathogenesis and Potential Implications as Clinical Biomarkers. Front. Mol. Neurosci..

[5] An H., Williams N.G., Shelkovnikova T.A. (2018). NEAT1 and paraspeckles in neurodegenerative diseases: A missing lnc found?. Noncoding RNA Res..

[6] Lin Y., Schmidt B.F., Bruchez M.P., McManus C.J. (2018). Structural analyses of NEAT1 lncRNAs suggest long-range RNA interactions that may contribute to paraspeckle architecture. Nucleic Acids Res..

[7] Yamazaki T., Souquere S., Chujo T., Kobelke S., Chong Y.S., Fox A.H., Bond C.S., Nakagawa S., Pierron G., Hirose T. (2018). Functional Domains of NEAT1 Architectural lncRNA Induce Paraspeckle Assembly through Phase Separation. Mol. Cell.

[8] Ma P., Li Y., Zhang W., Fang F., Sun J., Liu M., Li K., Dong L. (2019). Long Non-coding RNA MALAT1 Inhibits Neuron Apoptosis and Neuroinflammation While Stimulates Neurite Outgrowth and Its Correlation with MiR-125b Mediates PTGS2, CDK5 and FOXQ1 in Alzheimer’s Disease. Curr. Alzheimer Res..

[9] Masoumi F., Ghorbani S., Talebi F., Branton W.G., Rajaei S., Power C., Noorbakhsh F. (2019). Malat1 long noncoding RNA regulates inflammation and leukocyte differentiation in experimental autoimmune encephalomyelitis. J. Neuroimmunol..

[10] Li M., Wen S., Guo X., Bai B., Gong Z., Liu X., Wang Y., Zhou Y., Chen X., Liu L. (2012). The novel long non-coding RNA CRG regulates Drosophila locomotor behavior. Nucleic Acids Res..

[11] Martin J.R., Ollo R. (1996). A new Drosophila Ca^2+^/calmodulin-dependent protein kinase (Caki) is localized in the central nervous system and implicated in walking speed. EMBO J..

[12] Slawson J.B., Kuklin E.A., Ejima A., Mukherjee K., Ostrovsky L., Griffith L.C. (2011). Central regulation of locomotor behavior of Drosophila melanogaster depends on a CASK isoform containing CaMK-like and L27 domains. Genetics.

[13] Soshnev A.A., Ishimoto H., McAllister B.F., Li X., Wehling M.D., Kitamoto T., Geyer P.K. (2011). A conserved long noncoding RNA affects sleep behavior in Drosophila. Genetics.

[14] Soshnev A.A., Li X., Wehling M.D., Geyer P.K. (2008). Context differences reveal insulator and activator functions of a Su(Hw) binding region. PLoS Genet..

[15] Gummalla M., Maeda R.K., Castro Alvarez J.J., Gyurkovics H., Singari S., Edwards K.A., Karch F., Bender W. (2012). abd-A regulation by the iab-8 noncoding RNA. PLoS Genet..

[16] Li K., Tian Y., Yuan Y., Fan X., Yang M., He Z., Yang D. (2019). Insights into the Functions of LncRNAs in Drosophila. Int. J. Mol. Sci..

[17] Muraoka Y., Nakamura A., Tanaka R., Suda K., Azuma Y., Kushimura Y., Lo Piccolo L., Yoshida H., Mizuta I., Tokuda T. (2018). Genetic screening of the genes interacting with Drosophila FIG4 identified a novel link between CMT-causing gene and long noncoding RNAs. Exp. Neurol..

[18] Okamura K., Chung W.J., Ruby J.G., Guo H., Bartel D.P., Lai E.C. (2008). The Drosophila hairpin RNA pathway generates endogenous short interfering RNAs. Nature.

[19] Shimada S., Muraoka Y., Ibaraki K., Takano-Shimizu-Kouno T., Yoshida H., Yamaguchi M. (2020). Identification of CR43467 encoding a long non-coding RNA as a novel genetic interactant with dFIG4, a CMT-causing gene. Exp. Cell Res..

[20] Mohler J., Pardue M.L. (1982). Deficiency mapping of the 93D heat-shock locus in Drosophila melanogaster. Chromosoma.

[21] Sahu R.K., Mutt E., Lakhotia S.C. (2020). Conservation of gene architecture and domains amidst sequence divergence in the hsromega lncRNA gene across the Drosophila genus: An in silico analysis. J. Genet..

[22] Walldorf U., Richter S., Ryseck R.P., Steller H., Edstrom J.E., Bautz E.K., Hovemann B. (1984). Cloning of heat-shock locus 93D from Drosophila melanogaster. EMBO J..

[23] Garbe J.C., Pardue M.L. (1986). Heat shock locus 93D of Drosophila melanogaster: A spliced RNA most strongly conserved in the intron sequence. Proc. Natl. Acad. Sci. USA.

[24] Pardue M.L., Bendena W.G., Garbe J.C. (1987). Heat shock: Puffs and response to environmental stress. Results Probl. Cell Differ.

[25] Bendena W.G., Garbe J.C., Traverse K.L., Lakhotia S.C., Pardue M.L. (1989). Multiple inducers of the Drosophila heat shock locus 93D (hsr omega): Inducer-specific patterns of the three transcripts. J. Cell Biol..

[26] Mallik M., Lakhotia S.C. (2011). Pleiotropic consequences of misexpression of the developmentally active and stress-inducible non-coding hsromega gene in Drosophila. J. Biosci..

[27] Singh A.K., Lakhotia S.C. (2012). The hnRNP A1 homolog Hrp36 is essential for normal development, female fecundity, omega speckle formation and stress tolerance in Drosophila melanogaster. J. Biosci..

[28] Lakhotia S.C. (2011). Forty years of the 93D puff of Drosophila melanogaster. J. Biosci..

[29] Lakhotia S.C., Mallik M., Singh A.K., Ray M. (2012). The large noncoding hsromega-n transcripts are essential for thermotolerance and remobilization of hnRNPs, HP1 and RNA polymerase II during recovery from heat shock in Drosophila. Chromosoma.

[30] Prasanth K.V., Rajendra T.K., Lal A.K., Lakhotia S.C. (2000). Omega speckles—A novel class of nuclear speckles containing hnRNPs associated with noncoding hsr-omega RNA in Drosophila. J. Cell Sci..

[31] Singh A.K., Lakhotia S.C. (2015). Dynamics of hnRNPs and omega speckles in normal and heat shocked live cell nuclei of Drosophila melanogaster. Chromosoma.

[32] Hogan N.C., Traverse K.L., Sullivan D.E., Pardue M.L. (1994). The nucleus-limited Hsr-omega-n transcript is a polyadenylated RNA with a regulated intranuclear turnover. J. Cell Biol..

[33] Fini M.E., Bendena W.G., Pardue M.L. (1989). Unusual behavior of the cytoplasmic transcript of hsr omega: An abundant, stress-inducible RNA that is translated but yields no detectable protein product. J. Cell Biol..

[34] Hovemann B.T., Reim I., Werner S., Katz S., Saumweber H. (2000). The protein Hrb57A of Drosophila melanogaster closely related to hnRNP K from vertebrates is present at sites active in transcription and coprecipitates with four RNA-binding proteins. Gene.

[35] Jolly C., Lakhotia S.C. (2006). Human sat III and Drosophila hsr omega transcripts: A common paradigm for regulation of nuclear RNA processing in stressed cells. Nucleic Acids Res..

[36] Lo Piccolo L., Yamaguchi M. (2017). RNAi of arcRNA hsromega affects sub-cellular localization of Drosophila FUS to drive neurodiseases. Exp. Neurol..

[37] Chung C.Y., Berson A., Kennerdell J.R., Sartoris A., Unger T., Porta S., Kim H.J., Smith E.R., Shilatifard A., Van Deerlin V. (2018). Aberrant activation of non-coding RNA targets of transcriptional elongation complexes contributes to TDP-43 toxicity. Nat. Commun..

[38] Lo Piccolo L., Bonaccorso R., Attardi A., Li Greci L., Romano G., Sollazzo M., Giurato G., Ingrassia A.M.R., Feiguin F., Corona D.F.V. (2018). Loss of ISWI Function in Drosophila Nuclear Bodies Drives Cytoplasmic Redistribution of Drosophila TDP-43. Int. J. Mol. Sci..

[39] Fox A.H., Lamond A.I. (2010). Paraspeckles. Cold Spring Harb. Perspect Biol..

[40] Onorati M.C., Lazzaro S., Mallik M., Ingrassia A.M., Carreca A.P., Singh A.K., Chaturvedi D.P., Lakhotia S.C., Corona D.F. (2011). The ISWI chromatin remodeler organizes the hsromega ncRNA-containing omega speckle nuclear compartments. PLoS Genet..

[41] Sekido Y., Bader S.A., Carbone D.P., Johnson B.E., Minna J.D. (1994). Molecular analysis of the HuD gene encoding a paraneoplastic encephalomyelitis antigen in human lung cancer cell lines. Cancer Res..

[42] Corona D.F., Tamkun J.W. (2004). Multiple roles for ISWI in transcription, chromosome organization and DNA replication. Biochim. Biophys. Acta.

[43] Morcillo G., Diez J.L., Carbajal M.E., Tanguay R.M. (1993). HSP90 associates with specific heat shock puffs (hsr omega) in polytene chromosomes of Drosophila and Chironomus. Chromosoma.

[44] Alfonso-Parra C., Maggert K.A. (2010). Drosophila SAF-B links the nuclear matrix, chromosomes, and transcriptional activity. PLoS ONE.

[45] Zimowska G., Paddy M.R. (2002). Structures and dynamics of Drosophila Tpr inconsistent with a static, filamentous structure. Exp. Cell Res..

[46] Lakhotia S.C., Rajendra T.K., Prasanth K.V. (2001). Developmental regulation and complex organization of the promoter of the non-coding hsr(omega) gene of Drosophila melanogaster. J. Biosci..

[47] Mutsuddi M., Lakhotia S.C. (1995). Spatial expression of the hsr-omega (93D) gene in different tissues of Drosophila melanogaster and identification of promoter elements controlling its developmental expression. Dev. Genet..

[48] Johnson T.K., Cockerell F.E., McKechnie S.W. (2011). Transcripts from the Drosophila heat-shock gene hsr-omega influence rates of protein synthesis but hardly affect resistance to heat knockdown. Mol. Genet. Genomics.

[49] Singh A.K., Lakhotia S.C. (2016). Expression of hsromega-RNAi transgene prior to heat shock specifically compromises accumulation of heat shock-induced Hsp70 in Drosophila melanogaster. Cell Stress Chaperones.

[50] Fernandez-Funez P., Nino-Rosales M.L., de Gouyon B., She W.C., Luchak J.M., Martinez P., Turiegano E., Benito J., Capovilla M., Skinner P.J. (2000). Identification of genes that modify ataxin-1-induced neurodegeneration. Nature.

[51] Sengupta S., Lakhotia S.C. (2006). Altered expressions of the noncoding hsromega gene enhances poly-Q-induced neurotoxicity in Drosophila. RNA Biol..

[52] Mallik M., Lakhotia S.C. (2009). RNAi for the large non-coding hsromega transcripts suppresses polyglutamine pathogenesis in Drosophila models. RNA Biol..

[53] Mallik M., Lakhotia S.C. (2010). Modifiers and mechanisms of multi-system polyglutamine neurodegenerative disorders: Lessons from fly models. J. Genet..

[54] Mallik M., Lakhotia S.C. (2010). Improved activities of CREB binding protein, heterogeneous nuclear ribonucleoproteins and proteasome following downregulation of noncoding hsromega transcripts help suppress poly(Q) pathogenesis in fly models. Genetics.

[55] Brown R.H., Al-Chalabi A. (2017). Amyotrophic Lateral Sclerosis. N. Engl. J. Med..

[56] Kapeli K., Martinez F.J., Yeo G.W. (2017). Genetic mutations in RNA-binding proteins and their roles in ALS. Hum. Genet..

[57] Layalle S., They L., Ourghani S., Raoul C., Soustelle L. (2021). Amyotrophic Lateral Sclerosis Genes in Drosophila melanogaster. Int. J. Mol. Sci..

[58] Schwartz J.C., Wang X., Podell E.R., Cech T.R. (2013). RNA seeds higher-order assembly of FUS protein. Cell Rep..

[59] Lo Piccolo L., Attardi A., Bonaccorso R., Li Greci L., Giurato G., Ingrassia A.M.R., Onorati M.C. (2017). ISWI ATP-dependent remodeling of nucleoplasmic omega-speckles in the brain of Drosophila melanogaster. J. Genet. Genomics.

[60] Dormann D., Madl T., Valori C.F., Bentmann E., Tahirovic S., Abou-Ajram C., Kremmer E., Ansorge O., Mackenzie I.R., Neumann M. (2012). Arginine methylation next to the PY-NLS modulates Transportin binding and nuclear import of FUS. EMBO J..

[61] Hofweber M., Hutten S., Bourgeois B., Spreitzer E., Niedner-Boblenz A., Schifferer M., Ruepp M.D., Simons M., Niessing D., Madl T. (2018). Phase Separation of FUS Is Suppressed by Its Nuclear Import Receptor and Arginine Methylation. Cell.

[62] Lo Piccolo L., Mochizuki H., Nagai Y. (2019). The lncRNA hsromega regulates arginine dimethylation of human FUS to cause its proteasomal degradation in Drosophila. J. Cell Sci..

[63] Neumann M., Sampathu D.M., Kwong L.K., Truax A.C., Micsenyi M.C., Chou T.T., Bruce J., Schuck T., Grossman M., Clark C.M. (2006). Ubiquitinated TDP-43 in frontotemporal lobar degeneration and amyotrophic lateral sclerosis. Science.

[64] Mallik M., Lakhotia S.C. (2009). The developmentally active and stress-inducible noncoding hsromega gene is a novel regulator of apoptosis in Drosophila. Genetics.

[65] Lo Piccolo L., Jantrapirom S., Nagai Y., Yamaguchi M. (2017). FUS toxicity is rescued by the modulation of lncRNA hsromega expression in Drosophila melanogaster. Sci. Rep..

[66] Cooper T.A., Wan L., Dreyfuss G. (2009). RNA and disease. Cell.

[67] Han S.P., Tang Y.H., Smith R. (2010). Functional diversity of the hnRNPs: Past, present and perspectives. Biochem. J..

[68] He Y., Smith R. (2009). Nuclear functions of heterogeneous nuclear ribonucleoproteins A/B. Cell. Mol. Life Sci..

[69] Conlon E.G., Manley J.L. (2017). RNA-binding proteins in neurodegeneration: Mechanisms in aggregate. Genes Dev..

[70] Geuens T., Bouhy D., Timmerman V. (2016). The hnRNP family: Insights into their role in health and disease. Hum. Genet..

[71] Low Y.H., Asi Y., Foti S.C., Lashley T. (2021). Heterogeneous Nuclear Ribonucleoproteins: Implications in Neurological Diseases. Mol. Neurobiol..

[72] Nussbacher J.K., Tabet R., Yeo G.W., Lagier-Tourenne C. (2019). Disruption of RNA Metabolism in Neurological Diseases and Emerging Therapeutic Interventions. Neuron.

[73] Singh A.K., Choudhury S.R., De S., Zhang J., Kissane S., Dwivedi V., Ramanathan P., Petric M., Orsini L., Hebenstreit D. (2019). The RNA helicase UPF1 associates with mRNAs co-transcriptionally and is required for the release of mRNAs from gene loci. eLife.

[74] Chujo T., Yamazaki T., Kawaguchi T., Kurosaka S., Takumi T., Nakagawa S., Hirose T. (2017). Unusual semi-extractability as a hallmark of nuclear body-associated architectural noncoding RNAs. EMBO J..

[75] Jolly C., Konecny L., Grady D.L., Kutskova Y.A., Cotto J.J., Morimoto R.I., Vourc’h C. (2002). In vivo binding of active heat shock transcription factor 1 to human chromosome 9 heterochromatin during stress. J. Cell Biol..

